# Integrative and Comprehensive Pan-Cancer Analysis of Lymphocyte-Specific Protein Tyrosine Kinase in Human Tumors

**DOI:** 10.3390/ijms232213998

**Published:** 2022-11-13

**Authors:** Mingwei Han, Yiming Li, Yixiao Guo, Wanwan Zhu, Jianli Jiang

**Affiliations:** National Translational Science Center for Molecular Medicine, Department of Cell Biology, Fourth Military Medical University, Xi’an 710032, China

**Keywords:** LCK, TCGA, pan-cancer analysis, LIHC, LUAD

## Abstract

Lymphocyte-specific protein tyrosine kinase (LCK) is common in a variety of hematologic malignancies but comparatively less common in solid tumors. This study aimed to explore the potential diagnostic and prognostic value of LCK across tumors through integrative and comprehensive pan-cancer analysis, as well as experimental validation. Multiple databases were used to explore the expression, alteration, prognostic value, association with immune infiltration, and potential functional pathways of LCK in pan-cancers. The results were further validated by western blotting and qPCR of patient samples as well as tumor cell lines. High LCK expression typically represents a better prognosis. Notably, drug sensitivity prediction of LCK identified P-529 as a candidate for drug development. Gene Annotations (GO) and KEGG analyses showed significant enrichment of PD-L1 and the T-cell receptor pathway. The results from patient samples and tumor cell lines confirmed these conclusions in LIHC. In conclusion, LCK is differentially expressed in multiple tumors and normal tissues. Further analysis highlighted its association with prognostic implications, pan-cancer genetic alterations, and immune signatures. Our data provide evidence for a diagnostic marker of LCK and the possible use of LCK as a target for the treatment of tumors.

## 1. Introduction

The non-receptor tyrosine kinase LCK is a member of the Src kinase family that is expressed in all T lineage cells and is critically involved in cellular signal transduction as well as other important molecular processes [1]. Owing to the important role of LCK in immune cell responses, numerous studies have explored the functions and therapeutic values of LCK in hematologic malignancy [2,3,4,5,6]. The tyrosine kinase inhibitor dasatinib interferes with LCK and stops the cytolytic activity of T cells, which has been exploited to steer the activity of CAR T cells in real-time [7]. Studies have shown that 44.4% of childhood and 16.7% of adult T-cell acute lymphoblastic leukemia (T-ALL) cases respond well to dasatinib, and preTCR-LCK activation is the driver of dasatinib sensitivity [8].

However, the detailed biological role of LCK is still unknown, particularly in tumors. Several studies have detected LCK expression in many solid tumors, including lung, breast, and colon cancers [9]. Moreover, small-molecule inhibitors targeting LCK in human tumor cells have exhibited remarkable therapeutic effects [10,11]. As a typical tyrosine kinase, LCK comprises an SH3 domain in tandem, an SH2 domain at the amino terminal, and a kinase domain at the carboxy terminal [12]. The biological function of LCK differs depending on the cancer type [13]. Recent studies suggest that LCK may have tumorigenic and cancer-promoting functions in multiple tumors [9]. Jonas and co-workers identified LCK as a driver for the invasion and migration of oral cancer by exploiting tumor heterogeneity [14]. Moreover, LCK directly regulates the phosphorylation of TRPM8 and further accelerates the proliferation and migration of pancreatic cancer cells [15]. A study based on parallel genome-wide functional screens identified LCK as a key vulnerability to both proliferation and cisplatin resistance in nasopharyngeal carcinoma [16]. Paradoxically, in human breast cancer, LCK suppresses cellular invasion by decreasing MMP9, SKP2, and VEGF-A expression but promotes the metastasis of breast cancer [17]. Given the prominent therapeutic functions and recently accumulated evidence on tumors of LCK, an integrative and comprehensive pan-cancer analysis may help us further understand its functions in the development and progression of tumors.

**Figure 1:**
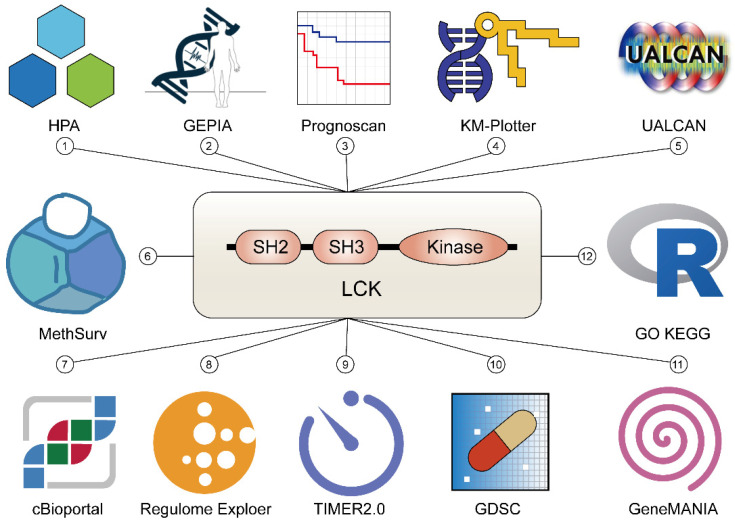
The schematic diagram shows the main databases and tools used in this study.

In this study, pan-cancer analysis based on a variety of databases was used to explore the role of LCK in human tumors, including their occurrence, development, progression, and potential signaling pathways (Figure 1). In particular, we explored the differential expression of LCK in TCGA tumors. The correlation between LCK expression and clinical outcomes was analyzed. Analyses of the expression levels, mutation information, and DNA methylation of LCK across different tumors were also conducted. Data from protein–protein interaction networks and Database for Annotation, Visualization, and Integrated Discovery (DAVID) were used for GO and KEGG enrichment analyses to further explore the potential functions of LCK in tumors. Immune reactivity and drug sensitivity analyses revealed the potential therapeutic value of LCK across tumors. Patient samples were used to detect LCK expression in tumor and normal tissues. The proliferative ability of the tumor cells was then detected using western blotting, qPCR, and flow cytometry.

## 2. Results

### 2.1. Expression Profiles of LCK in Different Human Tissues and Cells

**Figure 2:**
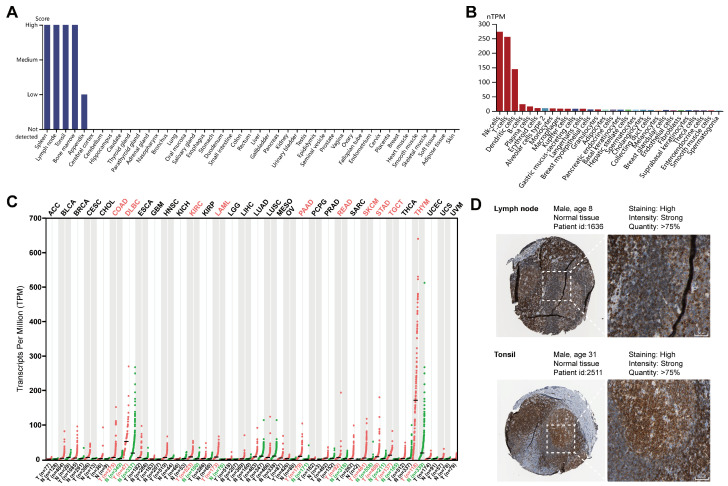
LCK expression profile across normal tissues and tumor samples. (**A**) Relative expression level of LCK in human normal tissues. (**B**) LCK expression was calculated using the consensus normalized expression (nTPM) value in different normal cell types. (**C**) Expression of LCK in 33 tumor tissues and their adjacent normal tissues. (**D**) Immunohistochemical staining of LCK in human lymph node and tonsil.

We first explored the LCK mRNA and protein expression levels in normal human tissues and cells. The HPA database showed that LCK was mainly expressed in the spleen, lymphoid tissues, and bone marrow but was rarely expressed in other tissues (Figure 2A and Figure S1). These results were further confirmed by LCK expression in the different cell types (Figure 2B). NK, T, and dendritic cells exhibited the highest expression of LCK in all normal cell types. Subsequently, we examined the expression of LCK across all tumor types and found that it was considerably elevated in various tumor types compared to normal tissues (Figure 2C), including lymphoid neoplasm diffuse large B-cell lymphoma (DLBC), kidney renal clear cell carcinoma (KIRC), acute myeloid leukemia (LAML), pancreatic adenocarcinoma (PAAD), rectum adenocarcinoma (READ), skin cutaneous melanoma (SKCM), stomach adenocarcinoma (STAD), testicular germ cell tumors (TGCT), and thymoma (THYM). LCK expression was significantly downregulated in patients with COAD, KICH, and LUSC (Figure S1B). The case numbers (Table S1) and clinical information (Table S2) of these tumors from TCGA are also summarized. Immunohistochemistry (IHC) from the HPA database further showed strong staining for LCK in the lymph nodes and tonsils (Figure 2D).

### 2.2. LCK Expression and Cancer Patient’s Prognosis

**Figure 3:**
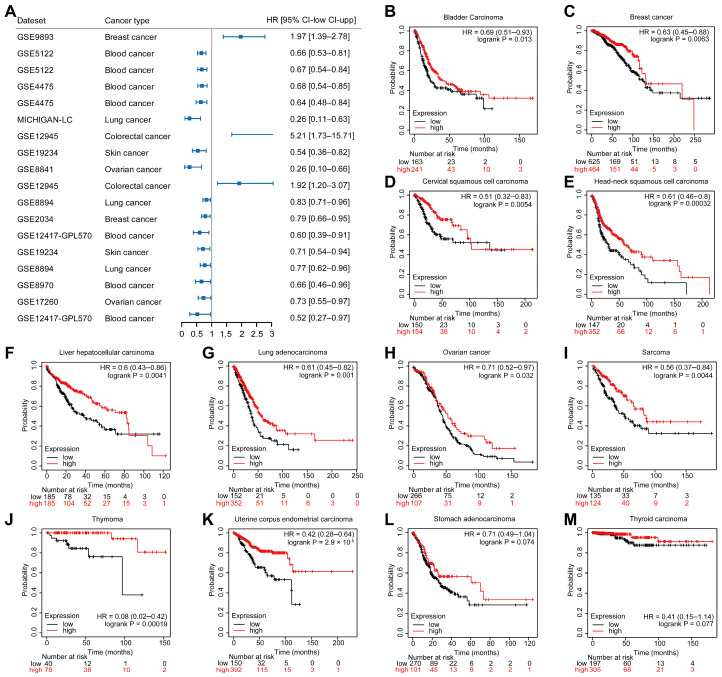
Prognostic value of LCK in different tumors. (**A**) Forest plot of LCK with several data cohorts from PrognoScan database. (**B**–**M**) Survival curves of LCK in different tumors from Kaplan–Meier Plotter.

LCK mRNA distribution showed relatively distinctive expression in tumors. The results from the PrognoScan database showed more distinct roles for LCK in the prognosis of different cancers. Several data cohorts (Figure 3A and Figure S2) revealed that high LCK expression was associated with favorable survival in patients with blood, lung, skin, and ovarian cancer, but it was poor in colorectal cancer. Further analysis using the Kaplan–Meier Plotter confirmed these results in multiple cancers (Figure 3B–M). Elevated LCK expression correlated with a better outcome among most human cancers including bladder carcinoma (HR = 0.69, 95% CI = 0.51–0.93, *p* = 0.013), breast cancer (HR = 0.63, 95% CI = 0.45–0.88, *p* = 0.0063), cervical squamous cell carcinoma (HR = 0.51, 95% CI = 0.32–0.83, *p* = 0.0054), head-neck squamous cell carcinoma (HR = 0.61, 95% CI = 0.46–0.8, *p* = 0.00032), liver hepatocellular carcinoma (HR = 0.6, 95% CI = 0.43–0.86, *p* = 0.0041), lung adenocarcinoma (HR = 0.61, 95% CI = 0.45–0.82, *p* = 0.001), ovarian cancer (HR = 0.71, 95% CI = 0.52–0.97, *p* = 0.032), sarcoma (HR = 0.56, 95% CI = 0.37–0.84, *p* = 0.0044), thymoma (HR = 0.08, 95% CI = 0.02–0.42, *p* = 0.00019), uterine corpus endometrial carcinoma (HR = 0.42, 95% CI = 0.28–0.64, *p* = 2.9 × 10^−5^), stomach adenocarcinoma (HR = 0.71, 95% CI = 0.49–1.04, *p* = 0.074), thyroid carcinoma (HR = 0.41, 95% CI = 0.15–1.14, *p* = 0.077). These results revealed that high LCK expression significantly correlated with good prognosis in most human cancers.

### 2.3. LCK Promoter Methylation and Prognostic Value of CpG Islands in the Survival of Tumor Patients

**Figure 4:**
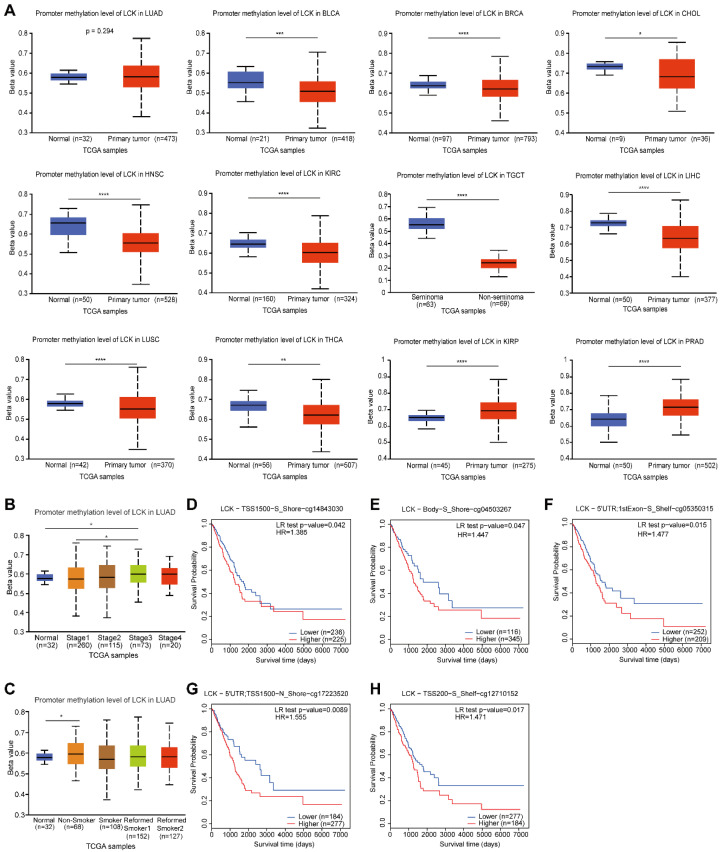
LCK DNA methylation in different tumors and its influence on the survival of patients with LUAD. (* *p* < 0.05, ** *p* < 0.01, *** *p* < 0.001, **** *p* < 0.0001). (**A**) The promoter methylation level of LCK in normal tissues and tumors across multiple tumor types. (**B**,**C**) The promoter methylation level of LCK in LUAD based on (**B**) sample stages and (**C**) smoking history. (**D**–**H**) The correlation between five CpGs methylation levels, (**D**) TSS1500-S_Shore-cg14843030, (**E**) Body-S_Shore-cg041503267, (**F**) 5′UTR; 1stExon-S_Shelf-cg05350315, (**G**) 5′UTR; TSS1500-N_Shore-cg17223520, and (**H**) TSS200-S_Shelf-cg12710152, and their effects on the overall survival evaluating by the Kaplan–Meier test module in MethSurv.

As a major epigenetic regulator in human cancer, promoter methylation leads to gene expression silencing, which occurs on cytosine nucleotides across CpG islands [18]. *LCK* promoter methylation levels were analyzed across human tumors using the UALCAN database. Interestingly, we found that across most tumor types with statistical differences (*p* < 0.05), *LCK* promoter methylation was significantly lower than that in normal tissues, except for KIRP and PRAD (Figure 4A). We further explored the promoter methylation level of *LCK* in LIHC based on tumor grade (Figure S3A) and stage (Figure S3B), and found that higher grades of tumors were usually associated with lower levels of promoter methylation in LIHC. The basic patient information for these tumors from TCGA-LIHC is summarized in Supplementary Table S3. In LUAD, the promoter methylation of *LCK* was higher in stage three tumors (Figure 4B) as well as in non-smokers than in normal tissues (Figure 4C). These results indicate the potential diagnostic value of *LCK* promoter methylation in several types of tumor patients.

Subsequently, the methylation of CpG islands and the promoter methylation status of *LCK* and their clinical outcomes were investigated. Gene transcript and variant data analysis displayed multiple sequence alignments (MSA) of *LCK* transcript variants 1–3 (Figure S3C). An evolutionary tree of *LCK* transcript variants was generated by maximum composite likelihood analysis and visualized using the MEGA software (Figure S3D). Using methpimer predictions, three CpG islands were identified in LCK transcript variants 1 and 2 and two in variant 3 (Figure S3E). A comprehensive analysis of the correlation between clinicopathological features and methylation levels of *LCK* was performed using Methsurv in LUAD. The heatmap for TCGA-LUAD using the MethSurv database showed the global methylation levels of *LCK* (Figure S3F). Hypermethylation of most CpG sites in LUAD was identified. Among all CpG sites, cg14843030 (TSS1500, S_Shore), cg04503267 (Body, S_Shore), cg05350315 (5′UTR, S_Shore), cg17223520 (5′UTR, N_Shore), and cg12710152 (TSS200, S_Shelf) significantly and positively correlated with the prognosis of LUAD (Figure 4D–H).

### 2.4. Genetic Alterations and Mutations of LCK in Pan-Cancer Analysis

**Figure 5:**
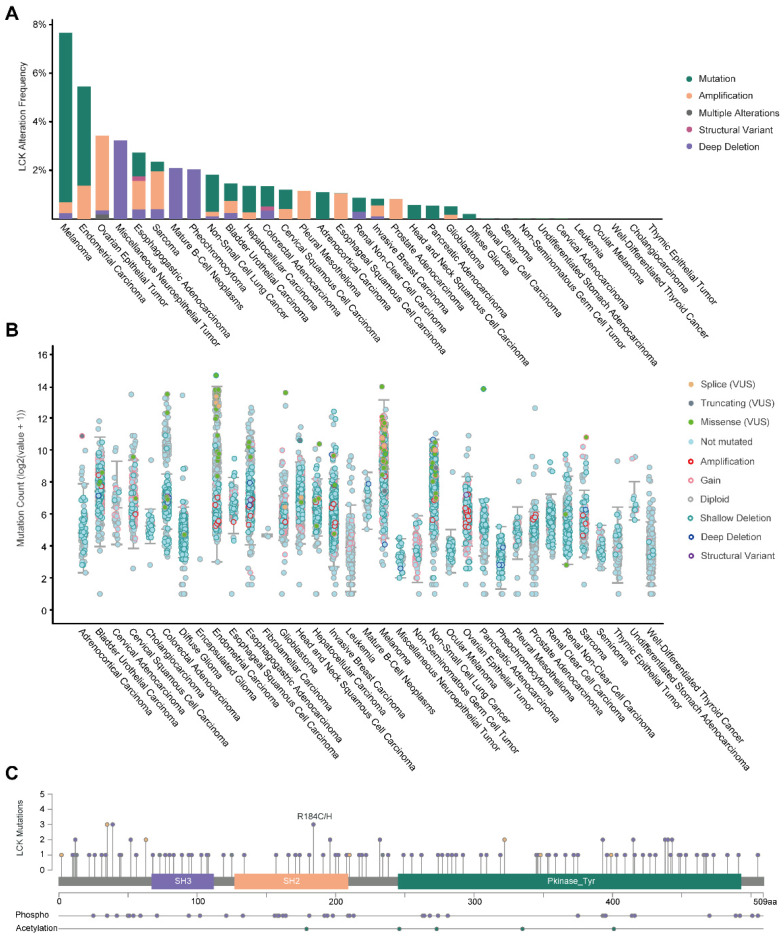
Genetic alteration types and protein mutations of LCK in different tumors. (**A**) LCK alteration frequency across different human tumors. (**B**) Mutation types and counts of LCK in human tumors. (**C**) Mutation diagram of LCK in different human cancers across protein domains.

Cancer-associated mutations in cancer genes constitute a diverse set of mutations associated with the disease [19]. To gain a deeper understanding of the genetic alteration characteristic of LCK across tumor types, we analyzed LCK alteration frequency using the cBioPortal online tool. The results revealed that patients with melanoma had the highest genetic alteration frequency of LCK (Figure 5A). Patients with ovarian epithelial tumors exhibited significant amplification of LCK. Figure 5B shows the alteration counts of the different tumors in detail.

Protein function is highly dependent on its structure, and proteins usually acquire new functions through mutations in their amino acid sequence during evolution [20]. We investigated the protein structure and mutations in the LCK gene using cBioPortal. The structure of LCK comprises three domains, including an SH3, an SH2, and a kinase domain (Figure 5C and Figure S4C). An R184C/H alteration in the SH2 domain was also detected. The COSMIC database provided more information about the mutation (Figure S4A) and substitution (Figure S4B) types of LCK. Missense mutations (41.45%) and G > A substitutions (39.41%) were the most common.

### 2.5. Genome-Wide Association of LCK in Cancers

**Figure 6:**
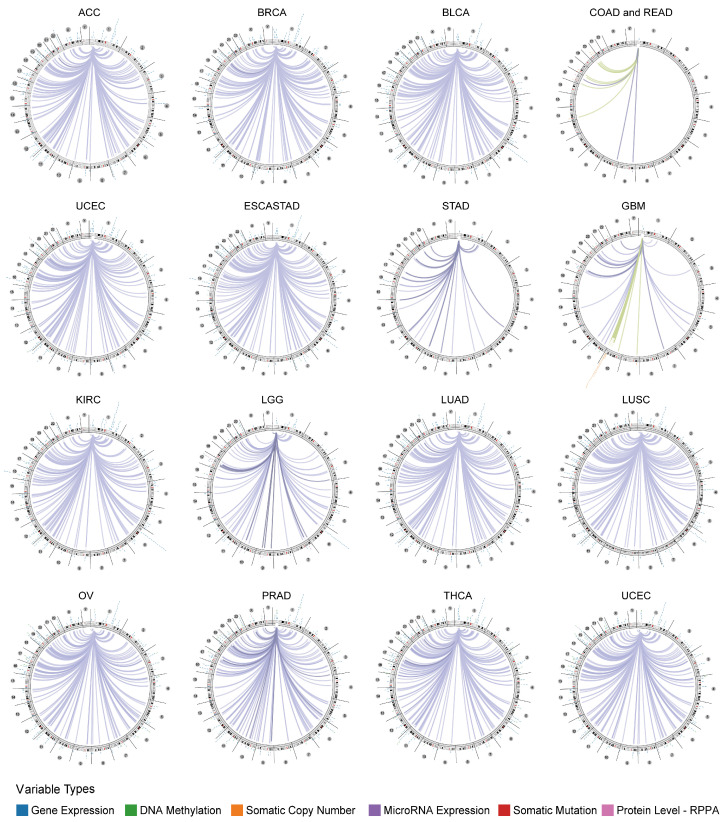
Genome-wide correlation analysis between *LCK* and other signatures of cancer samples from the Cancer Regulome program of The Cancer Genome Atlas database.

We explored the genomic association between *LCK* and certain signatures using the Regulome Explorer tool. Gene expression, DNA methylation, somatic copy number, microRNA expression, somatic mutation, and protein levels are shown using circus plots in different human cancers (Figure 6). In the circus plot, the circular layout edges were relevant, with the outer loop showing cytogenetic bands and the inner loop indicating an association with features lacking genomic coordinates [21]. Spearman’s correlation analysis correlates between a pair of genes (*p*-Value ≤ log10) using gene expression, DNA methylation, somatic copy number, miRNA expression, somatic mutation, and protein level data. According to data from the TCGA cancer regulome program, a large number of genes were significantly associated with *LCK* detected in ACC, BRCA, BLCA, UCEC, ESCASTAD (esophageal carcinoma, with gastric), STAD, KIRC, LGG, LUAD, LUSC, OV, PRAD, THCA, and UCEC. These results indicate that *LCK* is related to other genes in the genomes of these types of tumors.

### 2.6. Landscape of LCK Correlating with Immune Infiltration

**Figure 7:**
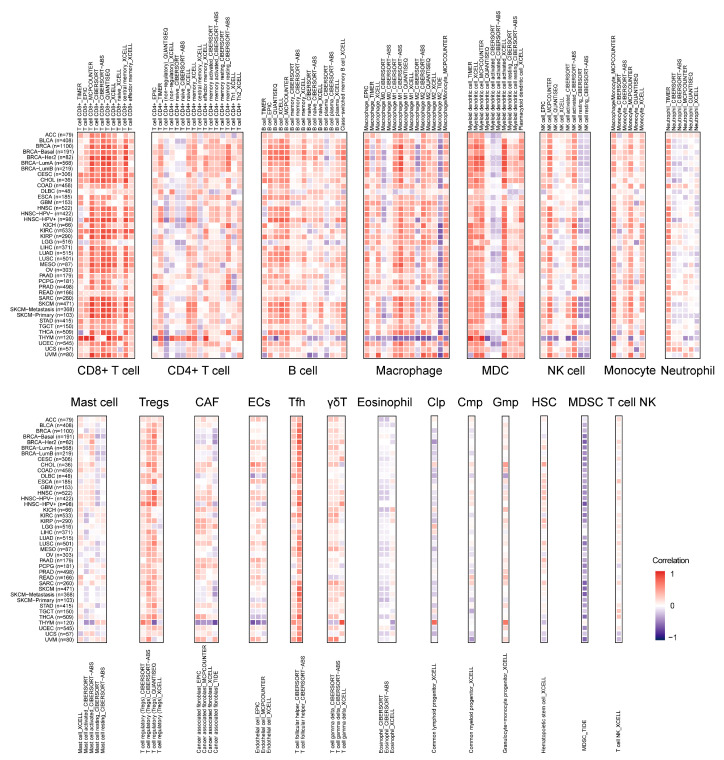
The correlations of LCK expression and immune infiltration across human cancers.

Tumor immune infiltrates are strongly associated with clinical features, invasion and metastasis status, and genetic alterations in cancer [22]. In the current study, TIMER2.0 was employed to explore whether LCK was involved in the process of immune infiltration. Interestingly, LCK positively correlated with the immune infiltrating levels of multiple immune cell types, especially CD8^+^ T cells (Figure 7). Nevertheless, a prominent negative correlation was observed between myeloid-derived suppressor cell (MDSC) infiltration and LCK expression. As an important protein kinase involved in immune signaling responses and regulation, our results further indicate that LCK may promote the infiltration of cytotoxic T cells and prevent MDSCs, which could suppress T cell functionality. In addition, tumor heterogeneity was observed, given that only THYM patient samples showed a significantly negative correlation between LCK expression and macrophage abundance. Further studies are required to clarify these differences.

### 2.7. Relationship between Immune Checkpoints and LCK

**Figure 8:**
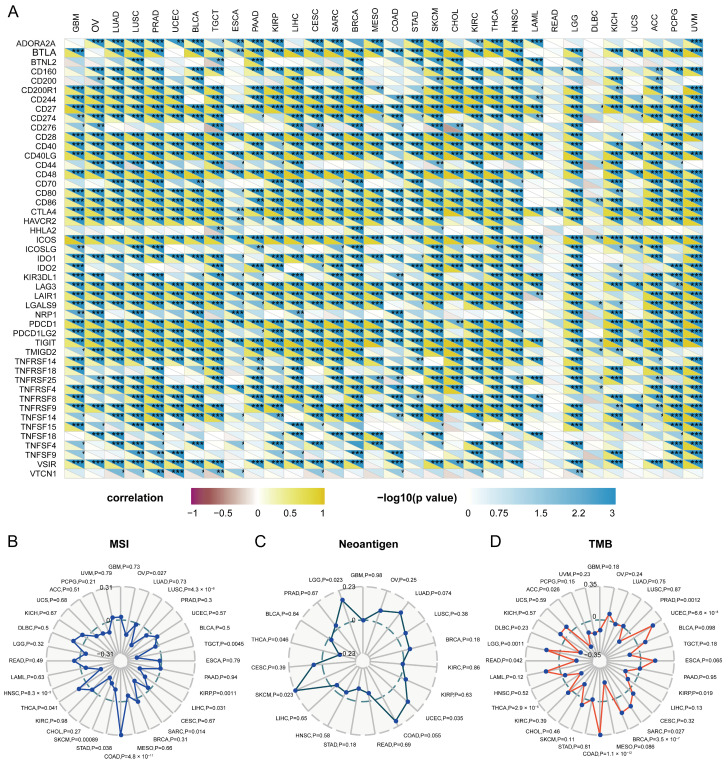
Correlations between LCK and immune checkpoints and other variables of interest. (* *p* < 0.05, ** *p* < 0.01, *** *p* < 0.001). (**A**) The correlations between LCK and confirmed immune checkpoints in multiple cancers. (**B**–**D**) The correlations of LCK expression and MSI, neoantigen, and TMB in tumors.

Next, we focused mainly on the relationship between LCK and immune checkpoints in different cancer types. Correlations were analyzed based on TIMER2.0 (Figure 8A). Significant positive correlations were observed between LCK expression and those of immune checkpoints, including B- and T-lymphocyte attenuator (BTLA), CD200R1, CD244, CD27, CD28, CD40, CD80, CD86, CTLA4 (cytotoxic T-Lymphocyte-associated antigen 4), inducible T cell costimulator, LAG3 (lymphocyte activating 3), PDCD1 (programmed cell death 1), and T cell immunoreceptor with Ig and ITIM domains (TIGIT). These results reveal the potential synergy of LCK with several immune checkpoints.

Microsatellite instability (MSI), the number of neoantigens, and tumor mutation burden (TMB) in tumor cells mainly cause genetic recombination and aggravation. The correlation between LCK expression and MSI events across different tumors was analyzed. LCK expression was positively associated with MSI in COAD (*p* < 0.001) and negatively associated with MSI in TGCT, HNSC, and LUSC (*p* < 0.01; Figure 8B). Moreover, patients with SKCM, UCEC, and LGG exhibited a significantly positive correlation between LCK expression and neoantigens (*p* < 0.05), whereas those with THCA, showed the opposite trend (*p* < 0.05; Figure 8C). Notably, positive correlations between LCK expression and TMB in COAD, UCEC, BRCA, LGG, and LAML (*p* < 0.001) were identified, whereas negative correlations were observed in ACC, KIRP, and PRAD (*p* < 0.05; Figure 8D).

### 2.8. Drug Sensitivity Analysis of LCK

**Figure 9:**
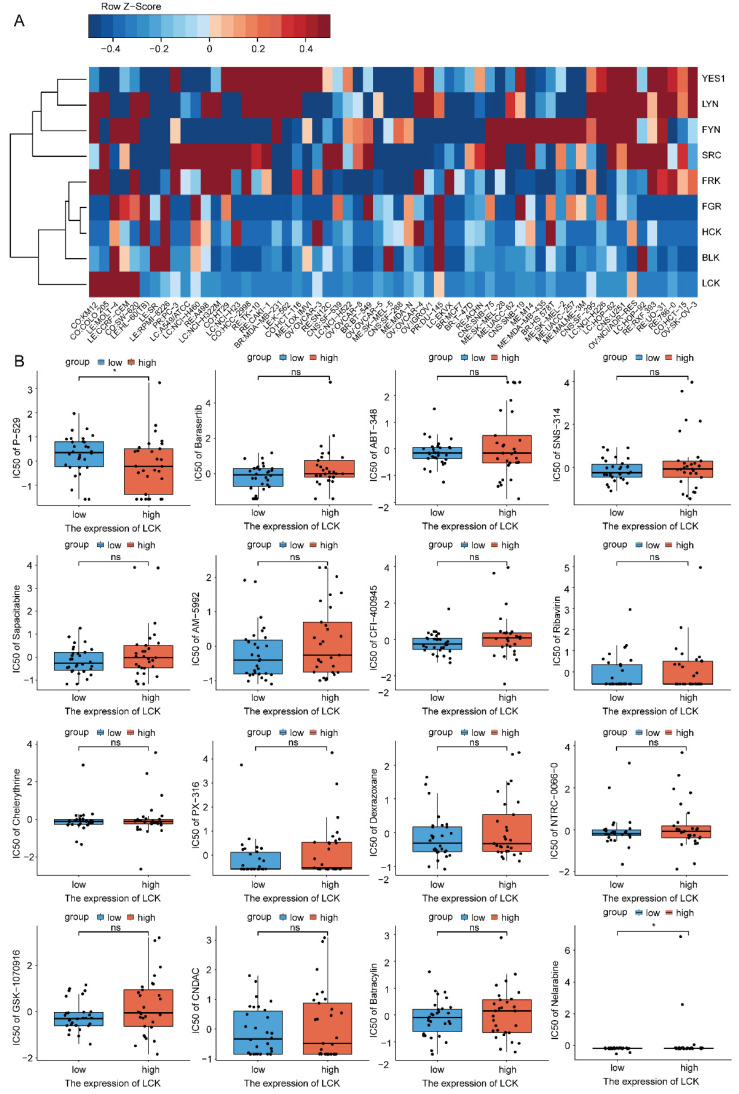
Drug sensitivity of LCK based on the CellMiner databases. (* *p* < 0.05). (**A**) The correlation between mRNA expression level of Src kinase family members and their corresponding z scores of cell sensitivity after drug treatment based on 60 cancer cell lines were exhibited with heatmap. (**B**) Box plots exhibited the top 16 correlations between LCK and drug sensitivity based on IC-50.

Given the encouraging drug targets of LCK in multiple cancers, we explored the potential inhibitors and diagnostic or therapeutic value of LCK using a drug discovery database. As the most widely used cancer cell sample group for anticancer drug testing, the 60 human cancer cell lines assembled by the National Cancer Institute (NCI-60) contain 22,379 confirmed genes and 20,503 analyzed compounds for anticancer drug discovery. In the “Download Data Sets” program, the “Processed Data Set” and “Compound activity: DTP NCI-60” were selected for further analysis (Table S4). LCK belongs to the SRC kinase family, including SRC, YES1, FYN, FGR, HCK, BLK, LYN, FRK, and LCK. The relationship between SRC kinase family members and drug sensitivity in NCI-60 cells was evaluated, and a sensitivity analysis of the correlation between LCK and drugs was also performed (Figure 9A). LCK exhibited relatively negative correlations with the multiple tested cell lines. We further evaluated the IC-50 values of multiple compounds by targeting LCK. To ensure the reliability of the results, we selected 574 clinical trials and 218 FDA-approved drugs; as a result, a total of 792 drugs were obtained and saved for subsequent analysis (Table S5). The correlation coefficients were calculated, and *p* < 0.01 was defined as the cut-off value for the results. Finally, from the specified top 16 components, we specified P-529 as a candidate for targeting LCK in tumors (Figure 9B) as it showed potent anti-proliferative activity against NCI-60 cell lines.

### 2.9. Protein–Protein Interaction Network and Pathway Enrichment of LCK

**Figure 10:**
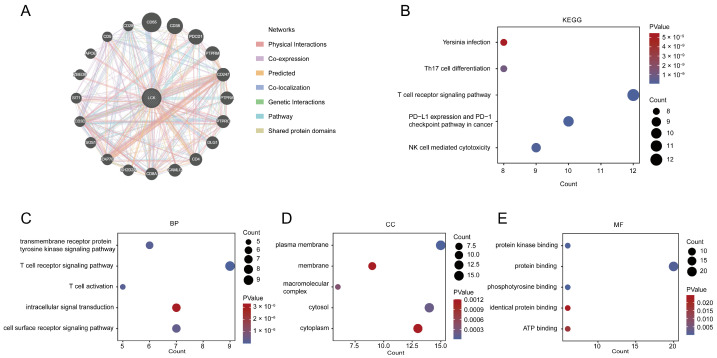
Protein–protein interaction network and pathway enrichment of LCK. (**A**) The proteins interacting with LCK based on the reported associations according to the GeneMANIA database. (**B**) KEGG enrichment analysis of LCK. (**C**–**E**) BP, CC, and MF analysis of LCK.

We also investigated the interacting proteins of LCK to illustrate their potential functions in tumors. GeneMANIA was used to explore the experimentally confirmed LCK interaction proteins, and subsequently, the interacting gene list was uploaded to the Database for Annotation, Visualization, and Integrated Discovery (DAVID) for enrichment analysis. KEGG and GO terms received from the database were further analyzed using the R package “ggplot2”. The results showed that CD55, CD38, PDCD1, PTPRM, CD247, PTPRA, and other proteins interacted directly with LCK (Figure 10A). KEGG and GO enrichment analyses showed that LCK was significantly associated with PD-L1 expression, T cell receptor signaling pathway, and PD-1 checkpoint pathway in cancers (Figure 10B). BP analysis showed a significant relationship between LCK and T cell receptor signaling pathways, and the main pathways were concentrated on T cell activation (Figure 10C). CC analysis showed that the plasma membrane, cytoplasm, cytosol, membrane, and macromolecular complexes were associated with LCK (Figure 10D). MF indicated the most significant enrichments in protein binding, protein kinase binding, phosphotyrosine binding, and ATP binding (Figure 10E).

### 2.10. Tumor Suppressor Role of LCK in Liver and Lung Cancer

**Figure 11:**
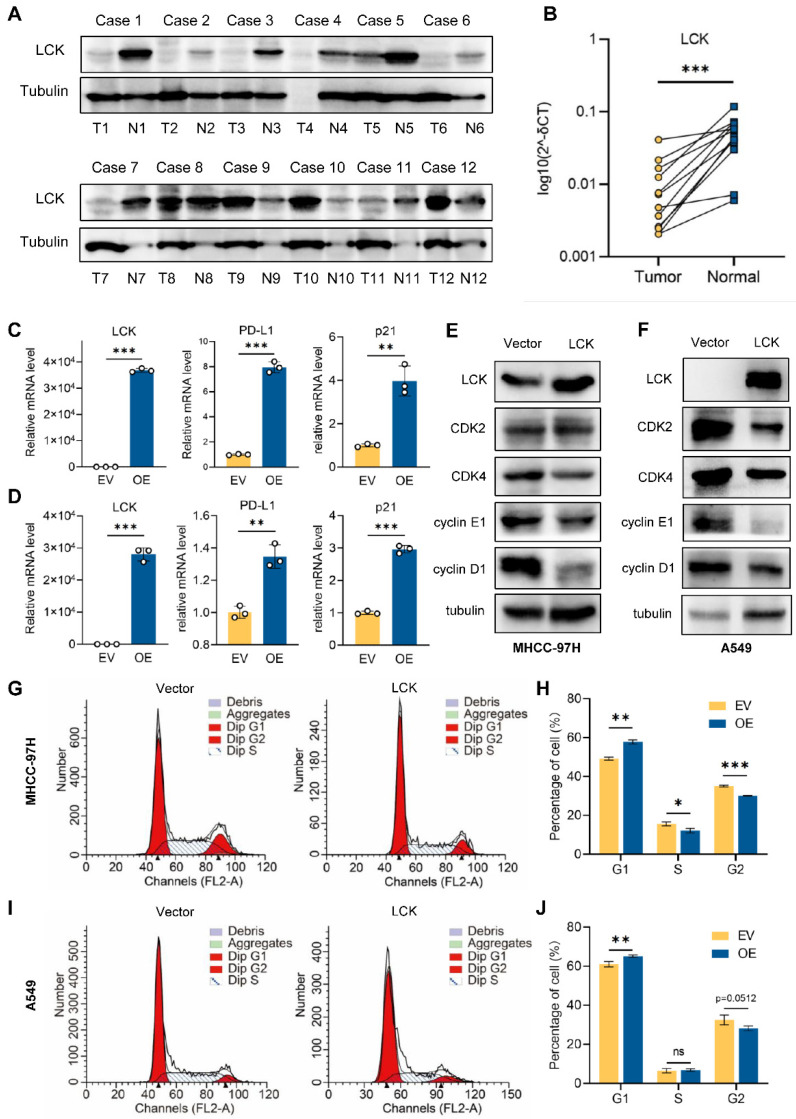
Downregulated expression of LCK in liver cancer and its potential influence on tumor cell cycle. (* *p* < 0.05, ** *p* < 0.01, *** *p* < 0.001). (**A**) Western blot analysis of LCK protein expression in 12 pairs of human LIHC and matched adjacent tissues. T, tumor; N, normal tissue. (**B**) qPCR analysis of LCK mRNA expression in 12 pairs of human LIHC and matched adjacent tissues. (**C**,**D**) Elevated mRNA expression level of PD-L1 and p21 after overexpression of LCK in MHCC-97H (**C**) and A549 (**D**) cells. (**E**,**F**) Decreased expression levels of cell cycle-related proteins, including CDK2, CDK4, cyclin E1, and cyclin D1, were identified with overexpression of LCK in MHCC-97H (**E**) and A549 (**F**) cells. (**G**–**J**) The cell cycle distribution was detected and quantified after LCK plasmid transfection in MHCC-97H (**G**,**H**) and A549 (**I**,**J**) cells.

To verify the expression and potential functions of LCK in tumors, 12 pairs of patient tissues and adjacent normal tissues of LIHC were used to identify LCK expression by western blotting (Figure 11A) and qPCR (Figure 11B). The results found that LCK was expressed at lower levels in LIHC samples than in normal liver tissues. KEGG and GO enrichment analyses (Figure 10B) suggested an association between LCK and PD-L1 expression. Consequently, a correlation analysis of LCK and PD-L1 based on GEPIA was conducted, which showed a positive association between LIHC (Figure S5A) and LUAD (Figure S5B). An increased mRNA level of PD-L1 was further observed following LCK overexpression in MHCC-97H (Figure 11C) and A549 (Figure 11D) cells.

LCK has been reported as a key factor regulating the functions of the cell cycle [23]; thus, several proteins related to the cell cycle were detected upon LCK overexpression (Figure 11C–F). We found increased mRNA levels of p21 and a significant decrease in cell cycle-promoting proteins such as CDK2, CDK4, cyclin E1, and cyclin D1, suggesting a possible tumor-suppressing role of LCK in liver and lung cancers. Cell cycle analysis further confirmed the cell cycle arrest in MHCC-97H (Figure 11G–H) and A549 (Figure 11I–J) cells following LCK overexpression. The discordant expression of PD-L1 and cell cycle-related proteins may indicate a subtype of tumor cells that feature a high proliferation ratio and low PD-L1 expression and may contribute to tumor metastasis, which implies both temporal and spatial heterogeneity of LCK and PD-L1 expression during metastatic progression [24].

## 3. Discussion

Previous studies have suggested that the biological functions of LCK are associated with T cell receptor signal transduction, T cell activation and development, cell survival, and apoptosis [25,26,27]. It is widely recognized that abnormal activation, expression, and transportation of LCK are significantly related to immune diseases in humans [28,29]. Robert J and co-workers showed that mislocalization of LCK impaired thymocyte differentiation and promoted thymoma development [30]; this brought forward LCK’s localization to specific cellular compartments, which is vital for its function. Moreover, studies on chronic lymphocytic leukemia (CLL) showed that LCK expression significantly correlated with the sensitivity of CLL cells to pharmacological treatment and may act as a potential therapeutic target for CLL patients [31,32].

LCK kinase activity of LCK is closely related to T-cell exhaustion, and according to recent studies, the heterogeneity within exhausted T cells largely results from immune checkpoint blockade permissive and refractory subsets such as stem-like and terminally differentiated cells [33]. This remarkable heterogeneity mainly marked tumorigenesis and treatment failure. A recent study showed that dasatinib, an LCK inhibitor, enhanced the anti-leukemia efficacy of CAR-T cells by inhibiting cell differentiation and exhaustion [34]. Tyrosine kinase inhibitors (TKI) targeting LCK exhibit powerful effects in reducing CAR-T cell differentiation and exhaustion, as well as enhancing therapeutic efficacy and in vivo persistence.

Classification of gastric cancer based on different immune signatures, such as altered LCK protein expression, predicted tumor responses to PD-1 inhibitors [35]. Moreover, in patients with cutaneous melanoma (CM), LCK has been associated with immune infiltration and survival benefits [36]. This evidence suggests that LCK likely functions not only by the intrinsic properties of cancer cells but also by the components in the tumor microenvironment (TME) [36]. The TME contains various types of immune cells, and their activation state is extremely important for influencing tumor progression and predicting prognosis, especially in cytotoxic CD8^+^ T cells. Apart from directly phosphorylating T cell antigen receptors, LCK can sense asparagine to promote CD8^+^ T cell activation through asparagine-mediated increases of LCK phosphorylation at tyrosine 394 (Y394) [2]. These findings highlight an unexpected role for LCK in the metabolic process of immune cells and the potential of targeting several nutrient metabolisms in both tumor and immune cells for tumor immunotherapy [37].

Protein kinases represent a highly dynamic and precisely regulated set of switches that control information and signal transduction in living organisms, for example, by transferring phosphate from adenosine triphosphate (ATP) to proteins [38]. As an important tyrosine kinase of the Src family, several studies on LCK have been conducted in solid tumors and have suggested its cancer-promoting functions [39,40,41,42,43,44,45]. For example, studies based on gene co-expression network analysis and the Gene Expression Omnibus database showed that LCK was upregulated in clear cell renal carcinoma and correlated with the promotion of tumor progression [46]. In addition, another study revealed that higher expression of LCK in muscle-invasive bladder cancer (MIBC) was associated with highly expressed immune checkpoints, such as CTLA4, PD-1, and PD-L1 [6]. Nonetheless, the detailed role of LCK in specific types of cancers remains unclear. Further gain-of-function and loss-of-function studies are needed to explore the function of LCK in different tumors.

The expression of LCK was significantly higher in tumor patients with COAD, DLBC, KIRC, LAML, PAAD, READ, SKCM, STAD, TGCT, and THYM than in control tissues in the RNA-Seq datasets GEPIA. These results suggest that LCK may have a tumor-promoting role in the occurrence, development, and progression of several types of tumors. The same results were obtained from biological experiments on several tumors and further validated by these conclusions from databases. However, given the limited research on tumor heterogeneity, LCK may play an inhibitory role in other tumors. In this study, decreased expression of LCK in liver cancer was identified, and a high expression of LCK was associated with favorable survival in patients with LIHC. Interestingly, the pan-cancer analysis also revealed a markedly favorable survival in tumors with high LCK expression, which indicated that LCK might participate in tumor growth inhibition.

Tumor mutation burden (TMB) is significantly correlated with responses to immune checkpoint inhibitors, but measuring tumor TMB by whole-exome sequencing (WES) is not clinically practical, and the definition of high TMB is inconsistent across clinical trials [35,47]. We demonstrated positive correlations between LCK expression and TMB in COAD, UCEC, BRCA, LGG, and LAML and negative correlations in ACC, KIRP, and PRAD. It was worth noting that these conclusions require to be supported by more robust experimental results. TMB has several strengths and intrinsic limitations as a biomarker in tumors and should be used carefully. Moreover, we analyzed the relationship between LCK and immune infiltration in different tumor types. Tumor-infiltrating lymphocytes (TILs) in the TME have been recognized as independent predictors of cancer patient prognosis and immunotherapeutic efficacy [48]. Our results showed a strong association between LCK and TILs, particularly CD8^+^ T cells. KEGG and GO analyses demonstrated that LCK was closely related to PD-L1 expression, which is considered an important immune checkpoint in antitumor immunity. Correlation analysis of LCK and PD-L1 based on GEPIA showed a positive relationship, which was further confirmed in lung and liver cancer cell lines. However, these data were derived from LCK overexpression. The lack of knockout data remains a limitation of the present study.

Tumor heterogeneity and cancer cell plasticity are often found in different types of human tumors and largely contribute to tumor progression and treatment failure. Although encouraging progress has been made by targeting LCK in in vitro and pre-clinical studies [49,50,51,52,53,54], it must be noted that many clinical trials using LCK inhibitors or pan-Src family kinase inhibitors have failed [55,56,57,58,59,60,61,62,63,64,65,66,67]. A possible reason for the failure of these clinical trials may be the multiple targeting of intracellular tyrosine kinases. Additionally, the tumor stage of the patient and their remission status also affect the outcome of treatment targeting LCK, which is a limitation of this research. The relationship between LCK expression and tumor stage in remission should be further investigated. Considering the “division of labor” across the Src-family tyrosine kinases, further studies may contribute to the development of more specific inhibitors targeting a single tyrosine kinase with fewer complications.

## 4. Materials and Methods

### 4.1. Expression Profiles of LCK in Human Normal and Tumor Tissues

The Human Protein Atlas (HPA) database (https://www.proteinatlas.org, accessed on 10 May 2022) includes protein expression levels in normal tissues, tumor tissues, and cells, as well as clinical information on tumor patients. We explored LCK mRNA distribution in normal human tissues and cells. In addition, the RNA-Seq dataset GEPIA (http://gepia.cancer-pku.cn/, accessed on 10 May 2022) was used to examine the expression levels of LCK in different tumor types. Based on The Cancer Genome Atlas (TCGA) and Genotype-Tissue Expression (GTEx), GEPIA provides key differential expression, correlation, and patient survival analyses, which creates opportunities for data mining and a deeper understanding of gene function [68]. Immunohistochemistry images of LCK protein were also collected from the tissue and pathology atlas panels of the HPA.

### 4.2. Prognostic Value of LCK in Patients with Tumors

The PrognoScan database (http://dna00.bio.kyutech.ac.jp/PrognoScan/index.html, accessed on 13 May 2022) was used to analyze the prognostic value of LCK in different tumors. This database has been recognized as a large collection of publicly available cancer microarray datasets with clinical annotation as well as a tool for assessing the biological relationship between gene expression and prognosis [69]. Hazard ratios (HR)and 95% confidence intervals (CI) were calculated for each study.

Kaplan–Meier Plotter (http://kmplot.com/analysis/, accessed on 15 May 2022) is an online web tool capable of assessing the correlation between gene expression and survival in 21 tumor types [70]. This database has been used to identify and validate survival biomarkers in tumors. Patients were divided into two groups (higher and lower expression levels according to the medium expression level) and compared using the Kaplan–Meier Plotter, and the prognostic value of LCK in multiple cancer types was explored.

### 4.3. LCK Promoter Methylation Level and CpG Sites on the Survival of Tumors

The nucleotide sequences of Homo sapiens LCK transcript variants 1 (NM 001042771.3), 2 (NM 005356.5), and 3 (NM 001330468.2) were downloaded from the National Center for Biotechnology Information (NCBI). Multiple sequence alignments (MSA) of the three variants were then compared. An evolutionary tree of LCK transcript variants was generated by the maximum composite likelihood analysis using MEGA 11.0.10 software. A CpG island was defined according to the following criteria: (i) at least 100 nucleotides, (ii) a GC percentage of at least 50%, and (iii) an observation/expectation CpG ratio of >0.6. As a portal to facilitate gene expression and survival analysis of tumor subgroups, UALCAN (http://ualcan.path.uab.edu/, accessed on 20 May 2022) allows users to perform DNA methylation of molecular profiles associated with multiple cancer types [71]. In this study, we explored the overall promoter methylation level of LCK and visualized it using the UALCAN database.

The MethSurv database (https://biit.cs.ut.ee/methsurv/, accessed on 20 May 2022) [72] is a web tool used to perform multivariate survival analysis using DNA methylation data. This database was used to explore CpG sites in the LCK gene, and the impact of their location on overall survival was examined through multivariable survival analysis. *p* < 0.05 was considered statistically significant.

### 4.4. Genetic Alteration Analysis of LCK across Tumors

The cBioPortal website (https://www.cbioportal.org/, accessed on 22 May 2022) is a tool for visualizing cancer genomic data analysis, and the data types include somatic mutations and DNA copy number alterations [73]. “TCGA Pan Cancer Atlas Studies” and “quick selection” tools on the website were used to explore the genetic alteration of LCK. The “mutations” module was used to exhibit the mutated site of the LCK gene. The COSMIC database (https://cancer.sanger.ac.uk/cosmic, accessed on 20 May 2022) was used to identify somatic mutations in LCK. This database represents the world’s largest and most comprehensive resource for exploring the impact of somatic mutations on human cancers.

### 4.5. Genomic Correlation of LCK Expression

Regulome Explorer (http://explorer.cancerregulome.org/, accessed on 9 May 2022) is a tool for exploring and understanding genomic association analysis, which shows the relationship between a gene and tumor genome according to the correlation among genes, DNA methylation, somatic copy number, somatic mutation, and protein level [74]. Circus plots were used to map genomic coordinates. Spearman correlations, associations with pairwise correlation ≥ 0.4 and −log10 (*p*-Value) ≥ 10 are shown in the circus plots.

### 4.6. Immune Infiltration Analysis

The Tumor Immune Estimation Resource 2.0 (TIMER2.0; http://timer.cistrome.org/, accessed on 2 June 2022) web server was used to assess the relationship between LCK and different tumor types of immune infiltration, which provided comprehensive analysis and visualization functions of tumor-infiltrating immune cells [75]. We evaluated the correlations between LCK and several infiltrating lymphocytes and assessed immune cell sets, including CD8^+^ T cells, CD4^+^ T cells, B cells, macrophages, and myeloid dendritic cells (MDC). Furthermore, the correlation between LCK expression and neoantigens, TMB, and MSI was investigated.

### 4.7. Discovering Drug Sensitivity of LCK in Tumor Cells

The Genomics of Cancer Drug Susceptibility (GDSC) database (www.cancerRxgene.org, accessed on 3 June 2022) provides significant information on molecular markers of drug sensitivity and response in cancer cells [76]. The GDSC data were used to identify the effects of LCK on drug sensitivity. In addition, the CellMiner database (https://discover.nci.nih.gov/cellminer/home.do, accessed on 3 June 2022) is a web-based suite of genomic and pharmacological tools that allows users to explore transcript and drug patterns in the NCI-60 cell line set [77]. We further evaluated the IC-50 of multiple screened compounds and mapped the top 16 compounds using R package “ggplot2”, and the function of stat_compare_means() based on R was used to calculate the *p*-value.

### 4.8. Protein–Protein Interaction and Pathway Enrichment of LCK

In-depth studies of protein–protein interactions play a critical role in discovering the molecular mechanism of protein functions [78]. Therefore, GeneMANIA (http://www.genemania.org, accessed on 6 June 2022) was used to explore the LCK protein–protein interaction networks, with the expectation of further investigating the function of LCK. The database for annotation, visualization, and integrated discovery (DAVID, https://david.ncifcrf.gov/, accessed on 6 June 2022) provides a comprehensive set of functional annotation tools for investigators to understand the biological meaning of large lists of genes. In the current study, DAVID was used to further analyze the potential biological processes (BP), cellular components (CC), molecular functions (MF), and Kyoto Encyclopedia of Genes and Genomes (KEGG) pathways of LCK. The top five significant pathways were visualized using R version 4.0.4.

### 4.9. Cell Culture

The human liver cancer cell line MHCC-97H and human lung cancer cell line A549 were obtained from the American Type Culture Collection (ATCC, Manassas, VA, USA) and cultured in DMEM and RPMI 1640, respectively, which contained 10% fetal bovine serum (FBS). All cells were maintained at 37 °C in a 5% CO_2_ atmosphere.

### 4.10. Transfection

The LCK overexpression plasmid was constructed (GeneChem, Shanghai, China) by cloning LCK into pcDNA3.1(+). The plasmid and its empty vector were transfected into MHCC-97H or A549 cell lines using jetPRIME (PT-114–15, Polyplus Transfection). After transfection for 30 h, cells were collected for further analysis.

### 4.11. Tissue Samples and Western Blotting

Twelve human liver carcinomas and adjacent normal tissues were obtained from the Tumor Tissue Bank of the National Translational Science Centre for Molecular Medicine. The tissues were obtained with informed consent, but clinical information was not available over time. Tissue samples were pretreated with TissueLyser II (Qiagen, Shanghai, China), and the supernatants were collected by centrifugation. Western blotting was performed with the corresponding primary antibodies (anti-LCK antibody, 2417S, CST; anti-CDK2 antibody, 10122-1-AP, Proteintech; anti-CDK4 antibody, 11026-1-AP, Proteintech; anti-cyclin E1, 11554-1-AP, Proteintech; anti-cyclin D1, 26939-1-AP, Proteintech; anti-tubulin, 66031-1-Ig, Proteintech, Wuhan, China). Horseradish peroxidase-conjugated anti-rabbit or mouse IgG (H + L) (Proteintech) was used to detect the primary antibodies and incubated at room temperature for 1 h. All antibodies were diluted using antibody dilution buffer (WB100D, NCM Biotech, Suzhou, China). Western blotting images were captured using a ChemiDocTMTouch Imaging System (Bio-Rad, Hercules, CA, USA).

### 4.12. RT-qPCR

Tissue and cell samples were collected, and total RNAs were retrieved using TRIzol reagent (Omega Bio-tek, Guangzhou, China). The RNAs were then reverse-transcribed to cDNA using the PrimeScript™RT reagent Kit (Takara, Otsu, Japan). After amplification with PCR (Bio-Rad), RT–qPCR was performed using the TB Green PCR kit (TaKaRa, Otsu, Japan) on the QuantStudio7 Real-Time PCR System (Thermo Fisher, Shanghai, China). Gene expression was measured based on β-actin expression. The primers for humans used in the reaction are as follows:

β-actin-Forward: CATGTACGTTGCTATCCAGGC,

β-actin-Reverse: CTCCTTAATGTCACGCACGAT;

LCK-Forward: TGCCATTATCCCATAGTCCCA,

LCK-Reverse: GAGCCTTCGTAGGTAACCAGT;

PD-L1-Forward: TGCCGACTACAAGCGAATTACTG,

PD-L1-Reverse: CTGCTTGTCCAGATGACTTCGG;

CDKN1A-Forward: AGGTGGACCTGGAGACTCTCAG,

CDKN1A-Reverse: TCCTCTTGGAGAAGATCAGCCG.

### 4.13. Cell Cycle Analysis

Cell cycle analysis of MHCC-97H and A549 cells was performed by flow cytometry. Briefly, cells were pretreated with 70% ethanol overnight at 4 °C. After centrifugation and washing with PBS three times; the cells were incubated with cell cycle analysis kit (Keygen, KGA512) reagents for 30 min at room temperature. The distribution of cell cycle phases and percentages was analyzed using a FACSCalibur Flow Cytometer (BD, Franklin Lakes, NJ, USA).

### 4.14. Statistical Analysis

GraphPad Prism 9.0 (San Diego, CA, USA) was used to perform the statistical analyses using unpaired Student’s *t*-test. All data were presented as mean ± SEM with at least three independent experiments. *p* < 0.05 was considered statistical significance.

## 5. Conclusions

In conclusion, LCK may play a critical role in immune cell signal transduction and serve as a potential prognostic and therapeutic biomarker in several cancers.

## Data Availability

All datasets used in this study are available from the corresponding author with reasonable request.

## Supplementary Materials

The following supporting information can be downloaded at: https://www.mdpi.com/article/10.3390/ijms232213998/s1.
