# Supplementary material for: Integrative and Comprehensive Pan-Cancer Analysis of Lymphocyte-Specific Protein Tyrosine Kinase in Human Tumors
Source: Int J Mol Sci. 2022 Nov 13;23(22):13998. doi: 10.3390/ijms232213998 (PMC9697346; doi:10.3390/ijms232213998)
Supplement: Supplementary file 1 [file ijms-23-13998-s001.zip › figureS1-5.pdf]

Supplementary material

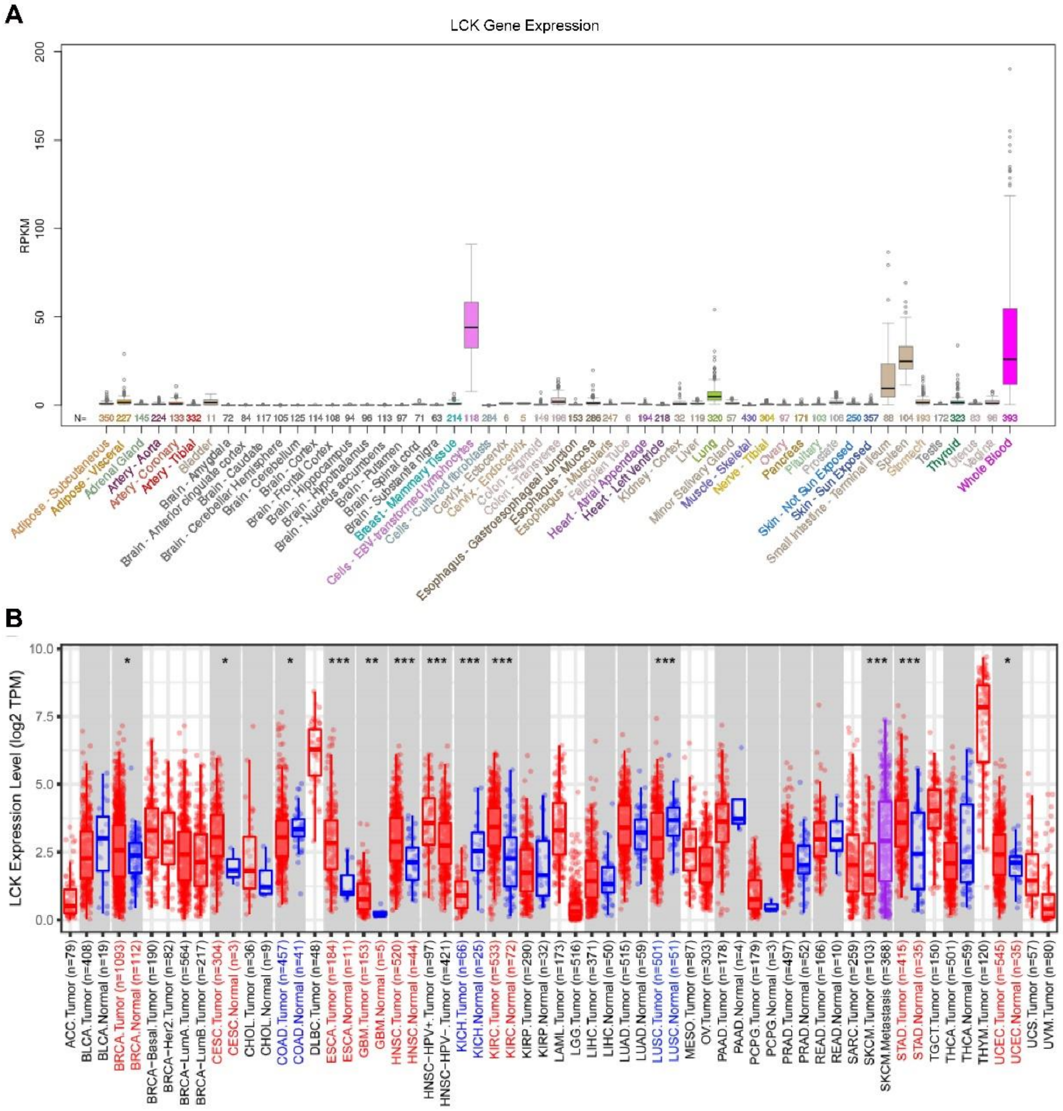

Figure S1. The expression of LCK in different tissues. The data used in the figure were obtained from GTEx Portal. (*p < 0.05, **p < 0.01, ***p < 0.001)

(A) LCK expression level according to tissue types.

(B) "Gene_DE" module of TIMER2.0 was used to study the differential expression between tumor and adjacent normal tissues for LCK across all TCGA tumors. Distributions of LCK expression levels were displayed using box plots. The statistical significance computed by the Wilcoxon test was annotated by the number of stars.

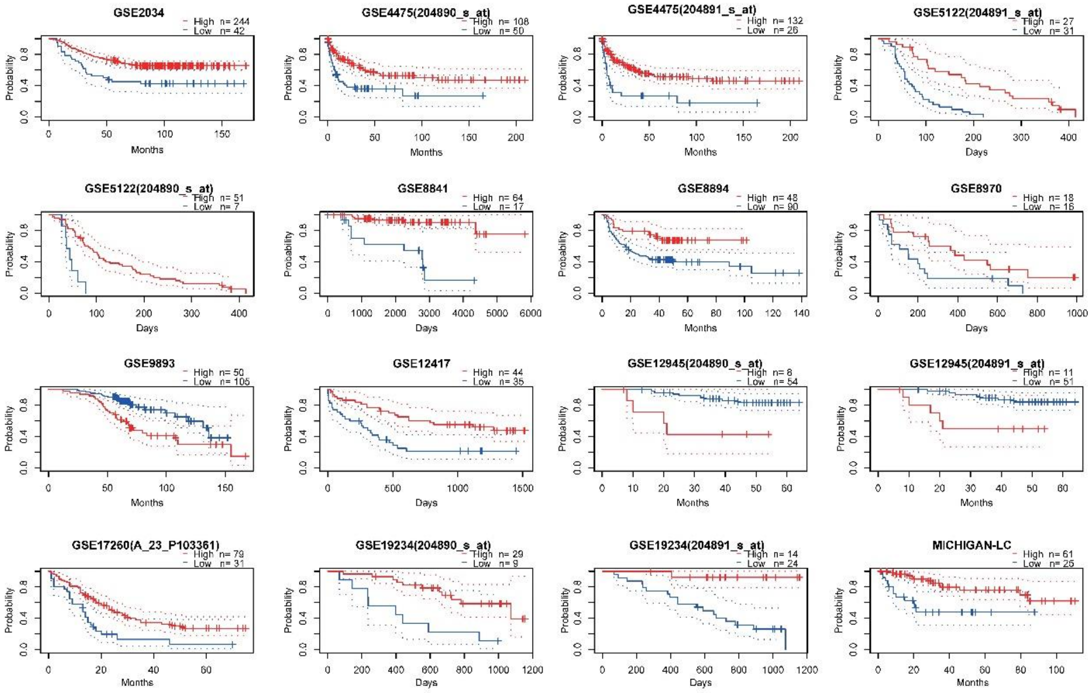

Figure S2. Prognostic value of LCK across tumors based on PrognoScan database.

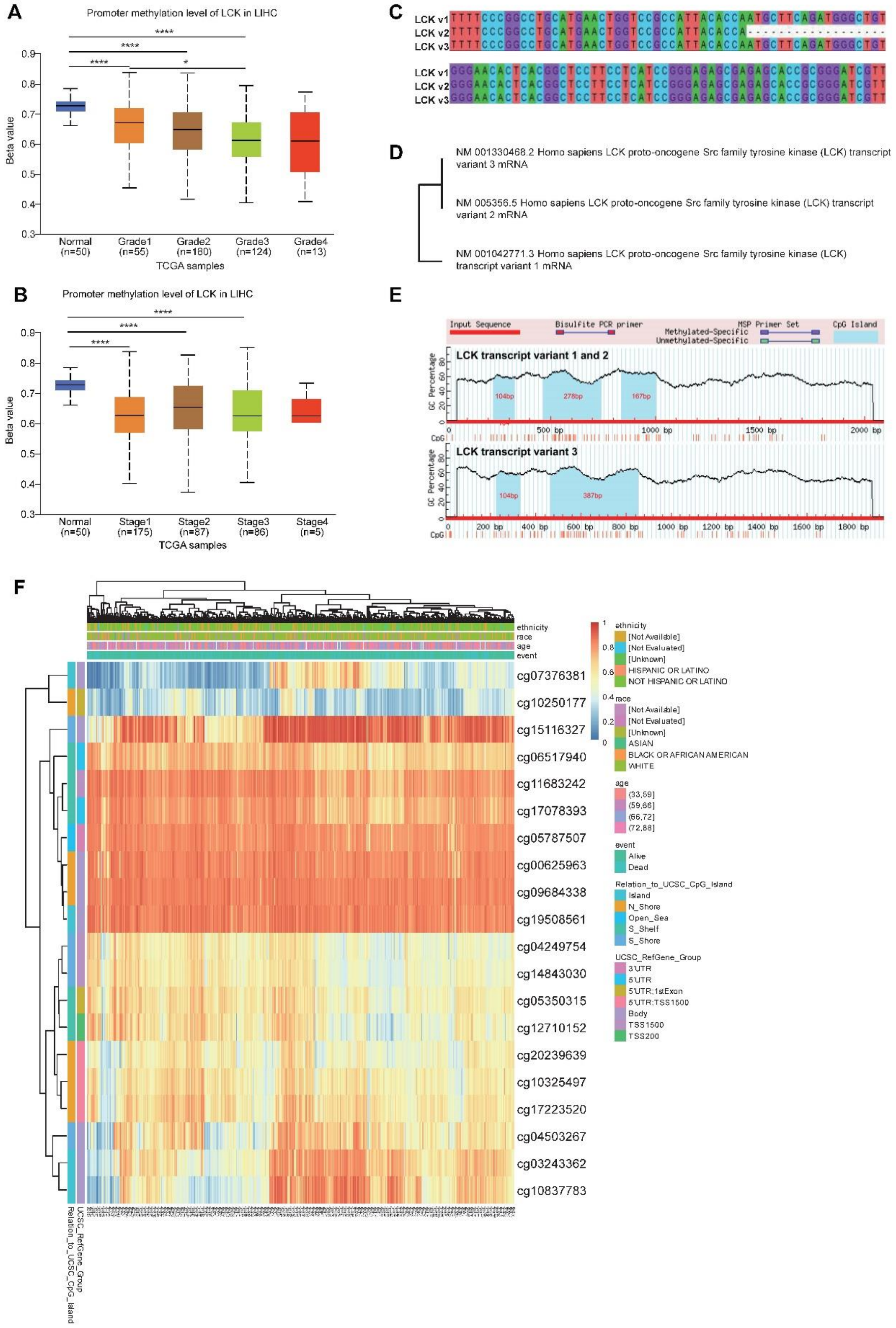

Figure S3. Promoter methylation level and CpG islands of *LCK*. (*$p < 0.05$, ****$p < 0.0001$)

(A, B) The promoter methylation level of LCK in LIHC based on grades (A) and sample stages (B). Compared with normal tissues, Grade 4 ($n = 13$) of LIHC samples exhibited lower LCK methylation level ($p < 0.05$).

(C) The multiple sequence alignments (MSA) of LCK transcript variant 1-3.

(D) An evolutionary tree of LCK transcript variants generated by the maximum composite likelihood analysis.

(E) Three CpG islands were identified in LCK transcript variant 1 and 2 and two in variant 3 by using Methpimer predictions.

(F) The heatmap demonstrated the global methylation levels of LCK in TCGA-LUAD. We correlated methylation levels with available patient characteristics and gene subregions by using the average linkage method with correlation distance. Methylation levels (1 = fully methylated; 0 = completely unmethylated) were shown as a continuous variable ranging from blue to red, and the row lines represent CpGs, and the columns correspond to the patients.

A

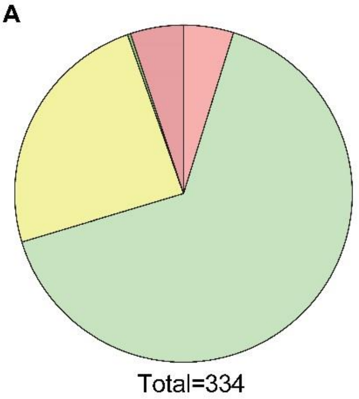

Color	Mutation type	Mutant samples	Percentage
	Nonsense substitution	16	3.04%
	Missense substitution	219	41.45%
	Synonymous substitution	81	15.37%
	Frameshift deletion	1	0.19%
	Other	17	3.23%
	Total unique samples	527	

B

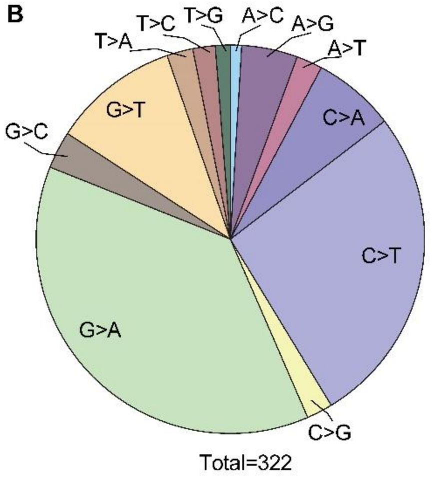

Color	Mutation	Number of samples	Percentage
	A>C	3	0.98%
	A>G	15	4.89%
	A>T	7	2.28%
	C>A	22	7.17%
	C>T	86	28.01%
	C>G	7	2.28%
	G>A	121	39.41%
	G>C	10	3.26%
	G>T	34	11.07%
	T>A	7	2.28%
	T>C	6	1.95%
	T>G	4	1.30%
Total unique samples		307	

C

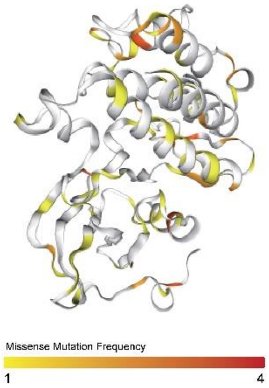

Figure S4. The distribution of different types of mutations for *LCK*.

(A) An overview of the types of mutation observed for *LCK*.

(B) A breakdown of the observed substitution mutations of *LCK*.

(C) The 3D structure of LCK protein.

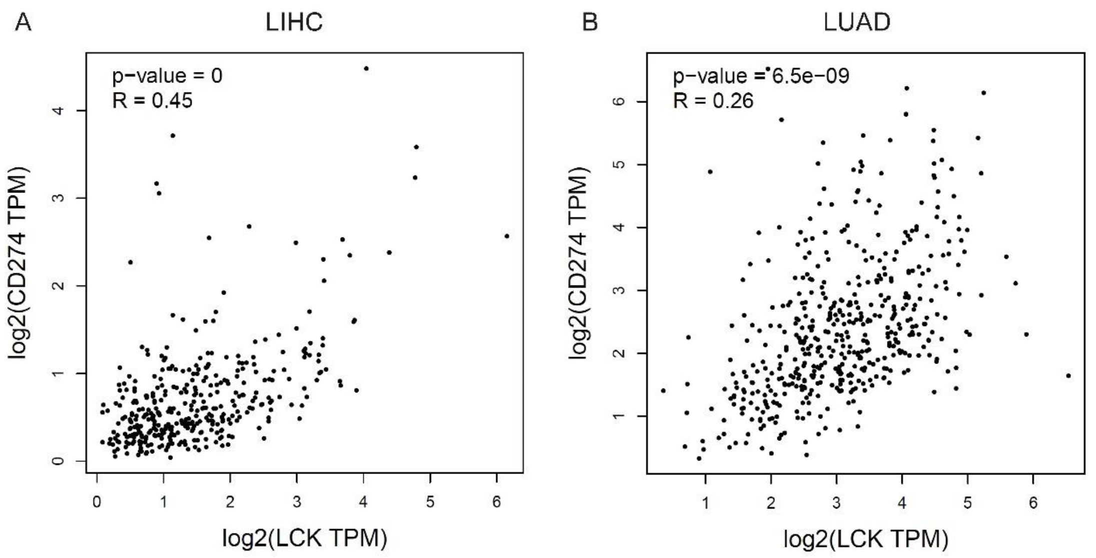

Figure S5. Correlation analysis of LCK and PD-L1 expression based on GEPIA in LIHC (A) and LUAD (B). The results were presented as scatter plots.
