# Supplementary material for: Integrative and Comprehensive Pan-Cancer Analysis of Lymphocyte-Specific Protein Tyrosine Kinase in Human Tumors
Source: Int J Mol Sci. 2022 Nov 13;23(22):13998. doi: 10.3390/ijms232213998 (PMC9697346; doi:10.3390/ijms232213998)
Supplement: Supplementary file 1 [file ijms-23-13998-s001.zip › Table S1-3.pdf]

Table S1. Case number of 33 tumor types from the cancer genome atlas

Cancer Type	Tumor Cases	Normal Cases	Cases
Adrenocortical Cancer (ACC)	92	5	97
Bladder Cancer (BLCA)	417	37	454
Breast Cancer (BRCA)	1121	163	1284
Cervical Cancer (CESC)	309	8	317
Bile Duct Cancer (CHOL)	51	20	71
Colon Adenocarcinoma (COAD)	478	93	571
Large B-cell Lymphoma (DLBC)	48	4	52
Esophageal Cancer (ESCA)	186	65	251
Glioblastoma (GBM)	631	40	671
Head and Neck Cancer (HNSC)	530	82	612
Kidney Chromophobe (KICH)	113	71	184
Kidney Clear Cell Carcinoma (KIRC)	543	442	985
Kidney Papillary Cell Carcinoma (KIRP)	293	88	381
Acute Myeloid Leukemia (LAML)	357	340	697
Lower Grade Glioma (LGG)	536	2	538
Liver Cancer (LIHC)	380	89	469
Lung Adenocarcinoma (LUAD)	603	274	877
Lung Squamous Cell Carcinoma (LUSC)	511	254	765
Mesothelioma (MESO)	87	1	88
Ovarian Cancer (OV)	623	135	758
Pancreatic Cancer (PAAD)	186	37	223
Pheochromocytoma Paraganglioma (PCPG)	184	5	189
Prostate Cancer (PRAD)	505	118	623
Rectal Cancer (READ)	173	19	192
Sarcoma (SARC)	266	24	290
Melanoma (SKCM)	474	3	477
Stomach Cancer (STAD)	443	101	544
Testicular Cancer (TGCT)	164	0	164
Thyroid Cancer (THCA)	515	100	615
Thymoma (THYM)	124	15	139
Endometrioid Cancer (UCEC)	554	52	606
Uterine Carcinosarcoma (UCS)	57	6	63
Ocular melanomas (UVM)	80	0	80

Table S2. Clinical information of tumors from the cancer genome atlas

Cancers	Samples	Age	Gender	Tumor Stage				Tumor Therapy	
		<60 (≥60)	Male (Female)	I	II	III	IV	Neoadjuvant	Radiation
ACC	92	68 (24)	32 (60)	9	44	19	18	1	9
BLCA	417	91 (326)	308 (109)	3	131	143	138	10	2
BRCA	1121	598 (522)	12 (1108)	186	637	253	20	13	14
CESC	309	243 (66)	0 (309)	163	70	47	22	0	4
CHOL	51	14 (31)	21 (27)	20	14	4	10	1	0
COAD	478	129 (347)	250 (226)	79	185	134	67	3	10
DLBC	48	26 (22)	22 (26)	8	17	5	12	1	6
ESCA	186	85 (101)	159 (27)	18	79	56	9	0	37
GBM	631	321 (300)	380 (241)	NA	NA	NA	NA	21	506
HNSC	530	237 (292)	387 (143)	27	74	82	272	10	17
KICH	113	85 (28)	62 (51)	54	33	19	7	1	0
KIRC	543	250 (293)	352 (191)	273	58	126	83	18	18
KIRP	293	122 (168)	215 (78)	174	22	52	15	0	5
LAML	357	184 (173)	195 (162)	NA	NA	NA	NA	40	0
LGG	536	465 (70)	293 (242)	NA	NA	NA	NA	3	64
LIHC	380	172 (207)	258 (122)	177	88	86	5	2	7
LUAD	603	144 (377)	253 (287)	293	126	85	28	67	67
LUSC	511	97 (405)	378 (133)	246	165	87	7	7	35
MESO	87	27 (60)	71 (16)	10	16	45	16	1	15

OV	623	324 (286)	0 (610)	18	33	465	89	1	5
PAAD	186	57 (129)	103 (83)	21	152	4	6	1	37
PCPG	184	142 (42)	82 (102)	NA	NA	NA	NA	1	2
PRAD	505	207 (298)	505 (0)	NA	NA	NA	NA	2	26
READ	173	54 (117)	93 (78)	33	51	52	25	1	23
SARC	290	118 (148)	121 (145)	NA	NA	NA	NA	1	51
SKCM	474	245 (221)	294 (180)	78	140	173	24	25	0
STAD	443	132 (306)	285 (158)	59	130	183	44	0	57
TGCT	164	137 (2)	139 (0)	56	12	14	NA	0	22
THCA	515	394 (121)	140 (375)	290	52	114	57	5	284
THYM	124	60 (63)	64 (60)	NA	NA	NA	NA	2	39
UCEC	554	181 (370)	0 (554)	NA	NA	NA	NA	3	16
UCS	57	6 (51)	0 (57)	NA	NA	NA	NA	0	11
UVM	80	36 (44)	45 (35)	0	39	36	4	0	3

NA: Not Available

Table S3. Patient information for LIHC from TCGA

Characteristics	Number of cases	Percentage (%)
Age		
<60	165	46.74
≥60	187	52.98
NA	1	0.28
Gender		
Male	242	68.56
Female	111	31.44
Clinical stage		
Stage I	175	49.58
Stage II	87	24.65
Stage III	86	24.35
Stage IV	5	1.42
Status		
Alive	236	66.86
Dead	117	33.14
Histologic grade		
G1	45	12.75
G2	172	48.72
G3	121	34.28
G4	13	3.68
NA	2	0.57
Metastases		
M0	269	76.20
M1	4	1.13
Mx	80	22.67
Cancer status		
Tumor free	155	43.91
With tumor	114	32.29
NA	84	23..80

TCGA: the cancer genome atlas; Cancer status: the state or condition of an individual's neoplasm at a particular point in time.
